# Rehabilitation in subjects with frozen shoulder: a survey of current (2023) clinical practice of Italian physiotherapists

**DOI:** 10.1186/s12891-024-07682-w

**Published:** 2024-07-23

**Authors:** Fabrizio Brindisino, Giuseppe Girardi, Mauro Crestani, Raffaele Assenza, Arianna Andriesse, Giuseppe Giovannico, Leonardo Pellicciari, Mattia Salomon, Davide Venturin

**Affiliations:** 1 Department of Medicine and Health Science “Vincenzo Tiberio”, University of Molise, Campobasso, Italy; 2 Department of Neurosciences, Biomedicine and Movement Sciences, University of Verona, Verona, Italy; 3 Physiotherapy Private Practice c/o Assenza Physical Therapy, Rome, Italy; 4 Medical Translation Private Practice c/o Andriesse Medical Translator, Rome, Italy; 5 IRCCS Istituto delle Scienze Neurologiche di Bologna, Bologna, Italy; 6 Department of Clinical Science and Translational Medicine, University of Roma “Tor Vergata”, Rome, Italy

**Keywords:** Frozen Shoulder, Shoulder Pain, Adhesive Capsulitis, Physiotherapy, Rehabilitation

## Abstract

**Objective:**

Frozen Shoulder (FS) is a musculoskeletal pathology that leads to disability, functional decline, and a worsening in quality of life. Physiotherapists are the primary professionals involved in the treatment of FS, and it is essential to determine if their practice aligns with evidence-based suggestions.

**Aim:**

The aim is to assess the knowledge, skills, and operational strategies of Italian physiotherapists regarding FS and compare them with the existing literature.

**Methods:**

A web-based, anonymous, and voluntary cross-sectional survey was developed and administered to Italian physiotherapists to evaluate their clinical practices.

**Results:**

A total of 501 physiotherapists (38.5% female), completed the survey. More than half were under 35 years old (67.8%), declared working in private practice settings or being self-employed (57.1%), and were primarily engaged with musculoskeletal patients (81.8%). For subjects with FS at their first access, 21.4% identified X-rays as the most useful imaging technique to recognize pathologies beyond rehabilitation competence. In terms of general management, the majority reported working with an orthopaedic or physiatrist (47.5%) or in a multidisciplinary team (33.5%). Regarding manual therapy techniques, 63.3% of physiotherapists preferred intense degree mobilization, posterior direction, and moderate pain at the end of the range of motion for low irritable/high stiffness FS; however, there is a lack of consensus for managing very irritable/low stiffness FS. The majority of physiotherapists (57.7%) concurred that stretching improves the balance between metalloproteinase and its inhibitors. Additionally, 48.3% of physiotherapists selected mobile phone videos and messages to improve patients’ compliance with exercises at home and for motivational/educational purposes.

**Discussion and Conclusion:**

The clinical practices of Italian physiotherapists in FS subjects sometimes deviate from evidence-based recommendations. While some discrepancies may be attributed to the existing uncertainties in the literature regarding knowledge and management strategies for FS patients, the authors recommend a stronger adherence to evidence-based practice.

**Supplementary Information:**

The online version contains supplementary material available at 10.1186/s12891-024-07682-w.

## Implications of Physiotherapy Practice

Italian PTs’ clinical practices in FS rehabilitation show partial alignment with evidence-based recommendations, particularly in the areas of diagnostic imaging suggestions and clinical assessment.

Italian PTs’ clinical practices exhibit partial adherence to evidence-based suggestions regarding the identification of predisposing factors, staging education, and mobilization modalities in FS cases.

Italian PTs with Orthopaedic Manipulative Physical Therapists (OMPT) certification, those possessing clinical experience ranging from 6 to 10 years, and individuals working in private practice demonstrate greater appropriateness in terms of knowledge, competence, and adherence to evidence-based clinical practices for FS rehabilitation.

**Supplementary Information:**

The online version contains supplementary material available at 10.1186/s12891-024-07682-w.

## Introduction

Frozen Shoulder (FS), also known as adhesive capsulitis [1, 2], is characterized by persistent, stabbing pain experienced both day and night, along with a gradual limitation of glenohumeral range of movement (ROM)—both active and passive—despite normal radiographic findings. Specific landmarks for diagnosis include a ROM restriction of at least 25% in at least two movement planes, with more than 50% limitation in external rotation at the arm by the side compared to the non-involved side. Additionally, the symptoms must be stable for at least one month or worsening [3, 4], persisting for a duration ranging from few months up to two years [5]. FS commonly affects individuals aged between 45 and 60 years, particularly those with sedentary jobs and low physical activity levels [3], and is often associated with comorbidities, such as diabetes, thyroid [6–11], autoimmune [7, 9], and Dupuytren’s disease [7, 8, 12].

Despite extensive study of the pathophysiology [13], the underlying mechanisms of FS remain unclear, leading to uncertainty about the optimal treatment. Evidence firstly suggested conservative treatment [13–15]; in particular, pharmacological treatments and physiotherapy, including education [16–18], active and passive glenohumeral mobilization [19, 20], stretching [2, 21], and therapeutic exercise [3] were favoured; while, electrotherapy was discouraged [22]. Given that FS significantly impacts quality of life, causing high disability and functional decline [21, 23], understanding the clinical practices of PTs becomes crucial, as they are the primary professionals involved in treating such pathologies. It is essential to assess whether their practice aligns with evidence-based recommendations.

Similar surveys conducted in the United Kingdom and the United Arab Emirates have investigated the diagnosis and management of FS among PTs [24, 25]. Thus, given the lack of such data in Italy, this study aimed to verify the knowledge, skills, and operative strategies of Italian PTs regarding FS treatment.

Evidence suggests that PTs with advanced competencies should be more likely to follow evidence-based practice in their clinical practice [26, 27]. Thus, Italian PTs with a postgraduate manual therapy specialization in Orthopaedic Manipulative Physiotherapy (OMPT) training were investigated to evaluate the effects of implementing evidence-based practice though such particular specialization course. Moreover, the present survey also assessed the impact of other professional characteristics that could condition the clinical practice as (1) years of working experience, (2) working context, (3) university education (recognized as the highest academic qualification), and (4) the number of patients with FS treated, on average, per month.

## Methods

### Study design

A web-based observational cross-sectional survey was conducted following the CHERRIES checklist [28] and the STROBE [29] reporting guidelines. The study protocol has been submitted and approved by the Technical Scientific Committee of the University of Molise (Italy). All study-related procedures were carried out following the principles of the Declaration of Helsinki [30].

### Participants and settings

The sample consisted of PTs practicing in Italy at the time of survey completion. The methodological approach employed in this survey, focused on attaining a maximum number of responses within a predetermined timeframe, is a conventional method for PTs enrolment [26, 27, 31, 32].

Survey participation was extended to professionals through various channels, including social media platforms (Facebook and Twitter), instant messaging applications (Telegram and WhatsApp), or email. The survey link directed PTs to a landing page emphasizing the voluntary and anonymous nature of their involvement. The consent declaration followed an explanation of the study’s purpose, stating, “The participant who willingly chooses to take part in the study must expressly grant consent by clicking the “YES” button, confirming acceptance”. Access to the survey was granted only upon clicking the button mentioned above, signifying consent acceptance (Appendix 1 – Invitation Letter). The respondent sample was categorized into two subgroups: colleagues with postgraduate OMPT specialization and colleagues without such specialization, facilitating meaningful comparisons. The OMPT title aligns with the Italian post-graduate program adhering to the International Federation of OMPT (IFOMPT) standards [33].

## Questionnaire development

An online survey was developed based on a questionnaire developed by Brindisino et al. [26]. This instrument was designed to evaluate the proficiency of Italian PTs across key domains: (a) clinical examination strategies, (b) the role of diagnostic imaging in the diagnostic process, (c) physiotherapy, and (d) pharmacological management for subjects with shoulder pain. Notably, the preceding survey targeted both surgeons and PTs; in contrast, the current questionnaire specifically focused on FS pathology and exclusively enlisted the participation of PTs. As a result, specific adjustments were implemented, notably the exclusion of questions related to pharmacological management. This modification reflects the legal limitations that prevent PTs in Italy from prescribing or administering drugs.

Furthermore, two experienced PTs, each with 12 and 15 years of expertise in rehabilitating shoulder pathologies, particularly stiff shoulders, contributed to the restructuring of certain questions. The authors specifically aimed to delve deeper into clinical examination procedures, incorporating six additional questions. Additionally, questions pertaining to the role of education, management strategies, and prognostic factors were introduced. A specific question was included to assess knowledge about the definition of FS. Other questions were tailored to comprehend how PTs evaluate and consider the patient’s perspective and engage in bio-psycho-social practices.

All these questions constituted the pre-final version of the survey. To enhance clarity and comprehensibility, the pre-final version underwent an evaluation by a team of colleagues with diverse experiences in shoulder disease rehabilitation. The team made modifications to only two questions and reached a consensus on the survey, resulting in the final version as follows. The first section (Appendix 2- Section A) consisted of 10 closed multiple-choice questions investigating demographic information for better framing the characteristics of respondents. PT’s year of work experience, working contest, University education (recognized as the highest academic qualification), having a post-graduate IFOMPT specialization degree, and the number of patients with FS treated, on average, per month, were also used to classify the respondents in sub-categories to perform inferences between groups.

The second section (Appendix 2 - Section B) included 22 specific closed multiple-choice questions concerning clinical examination knowledge and strategies, the role of imaging, physiotherapy management, definition, prognostic factors values and knowledge, and bio-psycho-social taking care approaches toward FS. Google Form was the online platform chosen for survey administration and data collection.

### Data collection

The survey was conducted using electronic devices and social media platforms and remained accessible for three months (from 01 April 2023 to 01 July 2023). The server was programmed to prevent multiple submissions from the same IP address after a successful submission. Respondents had the option to edit their answers by navigating through the survey questions until the final submission. Data collection was carried out anonymously, without recording IP addresses to safeguard respondent data. Subsequently, the data was forwarded to an external statistician, not involved in the study, for blind data analysis.

### Data analysis

Data extraction, processing, and addressing missing responses were performed using Excel. Questions with a missing response rate ≥ 20.0% were considered incomplete and excluded from the analysis [34]. Descriptive statistics, including mean ± standard deviation for interval variables and absolute frequencies with percentages for categorical variables, were used to represent the sample characteristics.

Statistical analysis to identify differences between subgroups of the sample and questionnaire responses utilized the chi-square test or Fisher’s exact test (for cell dimensions lower than 5). In cases where the Chi-Square (χ2) test revealed a statistically significant difference (*p* < 0.05), adjusted residuals with Bonferroni-corrected p-values were calculated [35] to determine the most contributing cells to the statistical significance of the study [36, 37]. The statistical analysis was conducted using SPSS version 20 Windows software (SPSS Inc., Chicago, Illinois, USA, 2004), with the α level set at p-value < 0.05.

### Role of funding source

The founders had no involvement in the design, conduct, or reporting of this study. Additionally, this research did not receive any specific grant from any funding agency in the public, commercial, or non-profit sectors.

## Results

### Descriptive information, overall sample

A total of 501 PTs completed the survey, and all questionnaires were filled out completely. The analysis revealed that a significant proportion of respondents were male (61.5%) under the age of 35 (67.8%), and 40.8% reported having less than five years of clinical experience. Notably, only 24.8% had clinical experience ranging from 6 to 10 years. Additionally, a majority of PTs indicated their affiliation with private practice or self-employment (57.1%), with a predominant focus on musculoskeletal patients (81.8%).

In terms of the number of FS patients treated, 74.5% of PTs reported managing two or fewer patients, on average, per month. Concerning educational background, 89.2% of PTs held a bachelor’s degree, and only 29.9% possessed a post-graduate IFOMPT specialization degree. The detailed descriptive characteristics of the sample are presented in Table 1.

**Table 1:** Descriptive demographic characteristics of the included subjects

Question	Multiple choice	Frequency (*N*)	Percentage (%)	C.I.
Sex **(Q2)**	Male	308	61.5	57.2–65.7
	Female	193	38.5	34.3–42.8
Working area **(Q3)**	Northern Italy	214	42.7	38.4–47.0
	Central Italy	124	24.8	21.0–28.5
	Southern Italy	163	32.5	28.4–36.6
Age **(Q4)**	≤ 25 years	70	14.0	10.9–17.0
	26–35 years	270	53.8	49.5–58.3
	36–45 years	93	18.6	15.2–22.0
	46–55 years	49	9.8	7.2–12.4
	≥ 56 years	19	3.8	2.1–5.5
Years of working experience **(Q5)**	≤ 5 years	205	40.8	36.6–45.2
	6–10 years	124	24.8	12.0–18.3
	11–15 years	76	15.2	21.0–28.5
	16–20 years	35	7.0	4.8–9.2
	≥ 21 years	61	12.2	9.3–15.0
Working contest **(Q6)**	Public Hospital	51	10.2	7.5–12.8
	Private structure/affiliated-accredited	141	28.1	24.2–32.1
	Private practice/self-employed activity	286	57.1	52.8–61.4
	Home-based activity	23	4.6	2.8–6.4
Area of work experience **(Q7)**	Musculoskeletal	410	81.8	78.5–85.2
	Sports	14	2.8	1.4–4.2
	Geriatric	34	6.7	4.6–9.0
	Neurology	29	5.8	3.7–7.8
	Other (Cardiological, Respiratory, Paediatric)	14	2.8	1.4–4.2
University education (highest Academic degree) **(Q8)**	Bachelor’s Degree in Physiotherapy	447	89.2	86.5–91.9
	Master’s Degree/Master of Science	52	10.4	7.7–13.0
	PhD	2	0.4	0.0–1.0
Post-graduate IFOMPT specialization degree (**Q9**)	Yes	150	29.9	25.9–34.0
	No	351	70.1	66.0–74.1
Average working hours per week **(Q10)**	0–10	9	1.8	0.6–3.0
	11–25	36	7.2	4.9–9.4
	26–35	119	23.7	20.0–27.5
	36–45	253	50.5	46.1–54.9
	≥ 46	84	16.8	13.5–20.0
Number of patients with FS, on average, per month **(Q11)**	≤ 2	373	74.5	70.6–78.3
	3	89	17.7	14.4–21.1
	4	19	3.8	2.1–5.5
	≥ 5	20	4.0	2.3–5.7

**Acronyms**: FS Frozen Shoulder, C.I. Confidence Interval, IFOMPT International Federation of Orthopaedic Manipulative Physical Therapist, N Number, PhD Doctor of Philosophy, Q Question

### Clinical knowledge and expertise of respondents (**Q12** - **Q33**)

Questions from **Q12** to **Q33** investigated the Italian PTs’ clinical knowledge and expertise (both for assessment and treatment) regarding managing subjects with FS.

When dealing with FS subjects at their first access (first assessment), in order to recognize pathologies beyond rehabilitation competence, 21.4% of PTs (**Q12**) identified X-ray as the most effective imaging technique. In contrast, 42.3% (**Q13**) believed that clinical examination alone, without additional imaging, was sufficient for the early identification of potential FS. A total of 39.1% of PTs (**Q14**) considered active and passive ROM assessment, clinical tests, and history-taking as the most valuable procedures for the early identification of FS.

The entire capsule or coracohumeral ligament was considered “the central pivot” of FS by 54.9% and 19.8% of respondents, respectively (**Q15**). In comparison, the most indicative clinical test for the diagnosis of FS was identified by 67.7% of PTs with a bilateral comparison of external rotation ROM with arm by side (**Q16**).

Sixty-four point five per cent of respondents defined FS following Kelley’s guideline [4] (**Q17**), and 48.7% recognized dysmetabolic diseases, age between 40 and 65, sedentary lifestyle, history of FS, being overweight, associated comorbidities as predisposing factors for FS (**Q18**). When considering priorities for patients, 32.3% and 19.6% of PTs emphasized the importance of reassurance about FS and night pain management, respectively (**Q19**). Additionally, 68.5% advocated tailoring the treatment based on the subject’s clinical presentation (**Q20**). Italian PTs expressed a consensus (89.6%) that education about the nature of the pathology, pharmacological interventions, and rehabilitative management should be integrated across the rehabilitation path, addressing both the psychological and pain management aspects (**Q21**).

In terms of pathology progression, PTs predominantly focused on educating individuals with FS by presenting it as a condition with either three detailed phases (39.5%) or two phases (28.7%) (**Q22**). They reported collaborating with physicians (47.5%) or functioning within multidisciplinary teams (33.5%), particularly when the expertise of other professionals is deemed necessary (**Q23**). Concerning the assessment (**Q24**), PTs universally considered both the pure anatomical aspect and the psychological aspects (fear, worry, anxiety, anger, distrust…) associated with the shoulder problem (85.6%). Furthermore, 88.4% of PTs expressed empathy and attentiveness to the psychological aspect of their clinical practice (**Q25**). The assessment of the psychological aspect mainly involved extemporaneous, individualized, and non-standardized questions (35.5%) (**Q27**).

The majority of PTs asserted that specific a-priori coded factors could indicate a worse prognosis (52.1%) (**Q26**). Additionally, 64.5% identified age < 60 years, external rotation at 0° adducted arm, diabetes and thyroid disease, bilaterality of clinical presentation, and worse symptoms at onset as factors suggesting a worse prognosis (**Q28**).

Regarding mobilization strategies (**Q29** and **Q30**), 63.3% of PTs expressed a preference for intense mobilization with a posterior direction, moderately painful at the end of the ROM for individuals with low irritability/high stiffness in FS. Conversely, for subjects with high irritability/low stiffness FS, 42.1% of PTs chose mobilization below the pain threshold in any direction, not very intense, at the end of the ROM. Additionally, 57.7% of PTs agreed that stretching improves the balance between metalloproteinase and tissue inhibitors of metalloproteinase (**Q31**). In terms of enhancing patients’ compliance with exercises at home and for motivational/educational purposes, 48.3% of PTs chose mobile phone videos and messages (**Q32**). Lastly, concerning conservative treatment (**Q33**), 53.5% of PTs stated that cortisone therapy (oral or infiltrative) is the preferred solution for better managing the painful phase. The detailed answers to questions **Q12-Q33** are reported in Table 2.

**Table 2:** Data analysis, clinical knowledge, and expertise (**Q12- Q 33**)

Question	Multiple choice	Frequency (*N*)	Percentage (%)	C.I.
Which type of imaging, among the following, do you think gives the best and most useful indications, at first access, in the patient with frozen shoulder/adhesive capsulitis to excising/tipping pathologies of non-rehabilitation competence? **(Q12)**	None, just the clinical examination	95	19.0	15.5–22.4
	X-ray	107	21.4	17.8–24.9
	MRI	96	19.2	15.7–22.6
	Ultrasound with Doppler	8	1.6	0.5–2.7
	X-ray and MRI	110	22.0	18.3–25.6
	MRI and Ultrasound with Doppler	59	11.8	9.0–14.6
	Ultrasound with Doppler and X-ray	26	5.2	3.2–7.1
Which type of imaging do you think gives the best and most useful indications, at first access, in the patient with frozen shoulder/adhesive capsulitis to identify a possible frozen shoulder/adhesive capsulitis early? **(Q13)**	None, just the clinical examination	212	42.3	38.0–46.6
	X-ray	19	3.8	2.1–5.5
	MRI	126	25.1	21.4–28.9
	Ultrasound with Doppler	42	8.4	6.0–10.8
	X-ray and MRI	37	7.4	5.1–9.7
	MRI and Ultrasound with Doppler	54	10.8	8.1–13.5
	Ultrasound with Doppler and X-ray	11	2.2	0.9–3.5
In your clinical practice, what do you believe gives better and more useful indications, at first access, in the patient with frozen shoulder/adhesive capsulitis to identify a possible frozen shoulder/adhesive capsulitis early? **(Q14)**	Anamnesis, X-ray, MRI	17	3.4	1.8–5.0
	Anamnesis, MRI, and clinical tests such as “coracoid pain test.”	23	4.6	2.8–6.4
	Anamnesis, physical examination of active and passive mobility, clinical tests such as “coracoid pain test.”	196	39.1	34.8–43.4
	Anamnesis, physical examination of active and passive mobility, clinical signs such as “capsular pattern.”	265	52.9	48.5–57.3
Which structure do you consider “the central pivot” (i.e., the structure that most often shows signs of pathology) of frozen shoulder/adhesive capsulitis? **(Q15)**	Long Head of the Biceps	26	5.2	3.2–7.1
	Coracohumeral ligament	99	19.8	16.3–23.2
	Tendon of the Supraspinatus	28	5.6	3.6–7.6
	The entire capsule	275	54.9	50.5–59.2
	Tendon of the Subscapularis	24	4.8	2.9–6.7
	None in particular	49	9.8	7.2–12.4
What is the most indicative clinical test for diagnosing frozen shoulder/adhesive capsulitis? **(Q16)**	Bilateral comparison of ROM in flexion	53	10.5	7.9–13.3
	Bilateral comparison of ROM in abduction	73	14.6	11.5–17.7
	Bilateral comparison of ROM in internal rotation	36	7.2	4.9–9.4
	Bilateral comparison of ROM in external rotation with the arm adducted	339	67.7	63.6–71.8
How best to define frozen shoulder/adhesive capsulitis? Which is the best definition of frozen shoulder/adhesive capsulitis? **(Q17)**	Shoulder pathology is characterized by night and day pain, with a reduction in active and passive range of motion, especially in abduction in the coronal plane, the changes in which must remain stable for at least one month	44	8.7	6.3–11.3
	Shoulder pathology is characterized by night and day pain at rest, with a reduction of active and passive range of motion, especially in external rotation with an abducted arm. Furthermore, changes must remain stable for at least one month or worsen.	71	14.2	11.1–17.2
	Shoulder pathology is characterized by night and day pain at rest and in motion, with a reduced active and passive range of motion, especially in external rotation with the arm adducted. Furthermore, changes must remain stable for at least one month or worsen.	323	64.5	60.3–68.7
	Shoulder pathology is characterized by night and day pain, with reduced active and passive range of motion, especially in sagittal plane flexion. Furthermore, the changes must remain stable for at least one month.	63	12.6	9.7–15.5
What are the predisposing factors for frozen shoulder/adhesive capsulitis? **(Q18)**	Dysmetabolic diseases, age between 40 and 65, sedentary lifestyle, previous frozen shoulder, overweight, neurological and cardiopulmonary comorbidities	245	48.7	44.3–53.1
	Age between 40 and 65, previous frozen shoulder, neurological and cardiopulmonary comorbidities	19	3.8	2.1–5.5
	Dysmetabolic diseases, age between 40 and 65, hyperactivity, female sex, comorbidity	218	43.5	39.2–47.9
	Dysmetabolic diseases, age between 50 and 60, male, musculoskeletal morbidity	19	3.8	2.1–5.5
	Dysmetabolic diseases, age between 40 and 65, sedentary lifestyle, previous frozen shoulder, overweight, neurological and cardiopulmonary morbidities, comorbidities	1	0.2	0.0–0.6
In your experience, what do you think is the priority for this type of patient: **(Q19)**	Daytime pain management	25	5.0	3.1–6.9
	Recovery of the full range of motion	26	5.2	3.3–7.1
	Nocturnal pain management	98	19.6	16.1–23.0
	Restorative sleep recovery	32	6.4	4.3–8.5
	Recovery of autonomy (driving, dressing…)	77	15.4	12.2–18.5
	Functional recovery related to work activities, hobbies, and social role	81	16.2	13.0–19.4
	Being reassured about one’s condition	162	32.3	28.2–36.4
In your clinical practice, which factors do you address the treatment’s characteristics (intensity, frequency, duration)? **(Q20)**	Level of daytime pain, level of stiffness, number of sessions	30	6.0	3.9–8.1
	Relationship between active/passive ROM, level of night and day pain	49	9.7	7.2–12.4
	Level of day and night pain, active/passive ROM ratio, presence of pain related to ROM	343	68.5	64.4–72.5
	Presence of pain related to range of motion, active/passive ROM ratio, presence of daytime pain	67	13.4	10.4–16.4
	Standard protocol for capsulitis	12	2.4	1.1–3.7
In your clinical practice, education on the nature of the pathology, its pharmacological and rehabilitative management: **(Q21)**	It is an aspect that I often overlook as not interesting/useful for the patient.	3	0.6	0.0–1.3
	It is a transversal intervention focused on the psychological component management throughout the rehabilitation process.	40	8.0	5.6–10.4
	It is a transversal intervention throughout the rehabilitation path, focused both on the psychological component and the pain portion management.	449	89.6	87.0–92.3
	It is an aspect I consider not very important for rehabilitation management.	9	1.8	0.6–3.0
In your clinical practice, according to the course of the pathology, you educate the patient with frozen shoulder/adhesive capsulitis: **(Q22)**	Evolution in 3 phases (in freezing-frozen-thawing), in detail	198	39.5	35.2–43.8
	Evolution in 2 phases (pain dominant or stiff dominant), in detail	144	28.7	24.8–32.7
	Evolution in 4 phases	22	4.4	2.6–6.2
	Evolution without specifying any phase	126	25.2	21.4–28.9
	With superficial explanations of this issue	6	1.2	0.2–2.2
	I do not consider it useful to provide this kind of explanation to the patient	5	1.0	0.1–1.9
In your clinical practice, you usually manage patients with frozen shoulder/adhesive capsulitis: **(Q23)**	Independently	89	17.8	14.4–21.1
	In cooperation with the doctor (orthopaedic, physiatrist)	238	47.5	43.1–51.9
	In collaboration with the psychologist	1	0.2	0.0–0.6
	In collaboration with the algologist	5	1.0	0.1–1.9
	In multidisciplinary teams, when the expertise of other professionals is required	168	33.5	29.4–37.7
In your clinical practice, you assess: **(Q24)**	Mainly anatomical aspects purely related to the shoulder problem (range of motion, pain, extent of stiffness)	44	8.8	6.3–11.3
	Ubiquitously, the anatomical aspect is purely linked to the shoulder problem and the psychological set-up (fear, worry, anxiety, anger, distrust…) related to the shoulder problem.	429	85.6	82.6–88.7
	The psychological rather than the anatomical aspect	9	1.8	0.6–3.0
	The anatomical rather than the psychological aspect	18	3.6	2.0–5.2
	Anatomical aspects purely related to the shoulder problem and the psychological set-up (fear, worry, anxiety, anger, distrust…) linked to the shoulder problem	1	0.2	0.0–0.6
In your clinical practice, when dealing with a patient with frozen shoulder/adhesive capsulitis, you tend to be predominantly: **(Q25)**	Empathetic and interested in building a relationship of trust	49	9.8	7.2–12.4
	Competent about the pathoanatomical condition, more than anything else	7	1.4	0.4–2.4
	Competent about the pathological condition, but equally empathic/attentive to the psychological set-up	443	88.4	85.6–91.2
	Exclusively focused on the pathological condition	2	0.4	0.0–1.0
Based on your knowledge about the patient’s prognosis with frozen shoulder/adhesive capsulitis, which of the following statements do you consider the most correct? **(Q26)**	The patient always recovers 100%, net of rehabilitation efforts and prognostic factors	82	16.3	13.1–19.6
	Rehabilitation is often ineffective and insufficient for optimal recovery and full patient satisfaction.	66	13.2	10.2–16.1
	The natural pathology course ends with a “restitutio ad integrum” without leaving any trace	92	18.4	15.0–21.8
	Coded factors that suggest to me, a priori, that the patient will be more unlikely to recover	261	52.1	47.7–56.5
In your clinical practice, how do you assess the psychological set-up of the patient with frozen shoulder/adhesive capsulitis: **(Q27)**	With validated measurement scales investigating catastrophizing, fear avoidance, anxiety, and depression	157	31.3	27.3–35.4
	With extemporaneous, subjectivized and non-standardized questions	178	35.5	31.3–39.7
	With a history interview	156	31.1	27.1–35.2
	I do not assess the psychological setting of the patient	10	2.0	0.8–3.2
What negative prognostic factors are identified in patients with frozen shoulder/adhesive capsulitis? **(Q28)**	Age > 60 years, thyroid disease, unilaterality of clinical presentation, less intense symptoms at onset	48	9.5	7.0–12.2
	Diabetes, hypothyroidism, external rotation at adducted arm > 0, more intense symptoms at onset	75	15.0	11.8–18.1
	Age < 60 years, external rotation at 0° adducted arm, diabetes and thyroid disease, bilaterality of clinical presentation, worse symptoms at onset	323	64.5	60.3–68.7
	Age, diabetes, hyperthyroidism, unilaterality of clinical presentation	55	11.0	8.2–13.7
In your clinical practice, what are the characteristics of the mobilization treatment of a patient with a frozen shoulder/adhesive capsulitis who is lowly irritable (MORE STIFF THAN PAINFUL)? **(Q29)**	Mobilization below the pain threshold, in any direction, not very intense, at the end of the range of motion	112	22.3	18.7–26.0
	Intense degree mobilization, posterior direction, moderately painful (approximately 6/10 NPRS) at the end of the range of motion	317	63.3	59.1–67.5
	Painful mobilizations in any direction, not very intense, not at the end of the range of motion	38	7.6	5.3–9.9
	Non-painful mobilizations, intense degree, not at the end of the range of motion, in posterior direction	34	6.8	4.6–9.0
In your clinical practice, what are the characteristics of the mobilization treatment of a patient with a very irritable frozen shoulder/adhesive capsulitis (MORE PAINFUL THAN STIFF)? **(Q30)**	Mobilization below the pain threshold, in any direction, not very intense, at the end of the range of motion	211	42.1	37.8–46.4
	Intense degree mobilization, posterior direction, moderately painful (approximately 6/10 NPRS) at the end of the range of motion	48	9.6	7.0–12.2
	Painless mobilization in any direction, not very intense, not at the end of the range of motion	189	37.7	33.5–42.0
	Non-painful mobilizations, intense degree, not at the end of the range of motion, in posterior direction	53	10.6	7.9–13.3
Which of the following statements about stretching do you agree with: **(Q31)**	Increases the centimetric length of fibrotic structures	123	24.6	20.8–28.3
	Improves the balance between metalloproteinase and tissue inhibitors of metalloproteinase	289	57.7	53.4–62.0
	Worsens tissue turnover	49	9.7	7.2–12.4
	Always increases pro-inflammatory expression	40	8.0	5.6–10.4
In your clinical practice, what strategies do you mainly use to increase patient compliance with exercises at home? **(Q32)**	Mobile phone videos and messages for motivational/educational purposes	242	48.3	43.9–52.7
	Illustrative booklet	96	19.2	15.7–22.6
	Diary	47	9.3	6.8–11.9
	None in particular	114	22.8	19.1–26.4
	I do not administer exercises at home	2	0.4	0.0–1.0
What do you mainly think is best associated with conservative treatment to manage the painful phase better: **(Q33)**	Physical therapy (laser therapy, diathermy, ultrasound, shockwaves)	91	18.2	14.8–21.5
	Cortisone therapy (oral or infiltrative)	268	53.5	49.1–57.9
	De-tensioning massage therapy	50	10.0	7.4–12.6
	Non-steroidal anti-inflammatories	92	18.3	15.0–21.8

**Acronyms**: C.I. Confidence Interval; MRI Magnetic Resonance Imaging; N Number; NPRS Numeric Pain Rating Scale; Q Question; ROM Range of Motion

### Subgroup analyses

This survey also evaluated the impact of (a) years of working experience, (b) working context, (c) university education (recognized as the highest academic qualification), (d) having a post-graduate IFOMPT specialization degree, and (e) the number of patients with FS treated, on average, per month. The responses to all clinical knowledge and expertise questions (**Q12-Q33**) were compared with the existing recommendations in the literature. To ensure consistency in the considered variables, categories were consolidated into new labels, as outlined in the “revised descriptive choices” presented in Table 3.

**Table 3:** Modified categories for subgroup analysis and inference

Question	Revised descriptive choices	Frequency (*N*)	Percentage (%)
Years of working experience **(Q5)**	≤ 5 years	205	40.9
	6–10 years	124	24.8
	> 10 years	172	34.3
Working contest **(Q6)**	Public sector	74	14.8
	Private structure/affiliated-accredited	141	28.1
	Private practice/self-employed activity	286	57.1
University education (highest academic qualification) **(Q8)**	Bachelor’s Degree in Physiotherapy	447	89.2
	Post Bachelor’s Degree	54	10.8
Number of patients with FS, on average, per month **(Q11)**	≤ 2	373	74.5
	≥ 3	128	25.5

**Acronyms**: FS, Frozen Shoulder; N, Number; Q, Question

### Years of work experience (**Q5**)

According to Bonferroni’s post-hoc analysis, PTs with 6 to 10 years of experience provided answers in accordance with evidence-based recommendations for **Q16**,** Q28**,** Q29**,** Q31**, and **Q33**. Contrariwise, PTs with more than ten years or with less than five years of experience significantly deviated from recommended practices in their answers to **Q14**,** Q29**,** Q31**,** and Q21**,** Q33**, respectively.

In terms of the correlation between years of working experience and **Q22**, where there was no recommended answer, a statistically significant analysis showed differences between groups for respondents with less than five years of experience and those with more than ten years of experience when FS was explained as a pathology with a 4-phase evolution.

### Working contest (**Q6**)

According to Bonferroni’s post-hoc analysis, PT engaged in private practice or self-employed activities adhered significantly to the recommendations when answering **Q32** and **Q33.**

Moreover, they are also less inclined to prioritize reducing daily pain in individuals with FS. Substantial differences between groups emerged in **Q19** and **Q22**, questions for which recommended answers were not outlined in the literature.

### University education (**Q8**)

According to Bonferroni’s post-hoc analysis and considering significant differences between groups and **Q25**, respondents with a post-bachelor degree tended to be more competent about the anatomical condition than all the other aspects. Furthermore, a significant difference was found between recommended answers to **Q15** and **Q26** and such group.

### Post-graduate IFOMPT specialization degree (**Q9**)

Statistical significance was obtained between respondents with IFOMPT certification and reassurance about the pathology as the priority for subjects with FS (**Q19**) and explaining the FS evolution as two phases (i.e., pain predominant or stiff predominant) (**Q22**). Moreover, such respondents were less inclined to solely assess anatomical features linked to FS (**Q24**) and significantly answered as recommended by evidence to questions **Q12**, from **Q15** to **Q18**, **Q20**, **Q21**, and from **Q27** to **Q33**.

### Number of patients with FS treated, on average, per month (**Q11**)

Respondents treating more than three subjects with FS per month were more likely to answer that ROM recovery was the priority for FS patients (**Q19**). Conversely, a significant positive association was found between the recommended answer to **Q28** and PTs who treated ≤ 2 patients with FS.

Bonferroni’s post-hoc analysis values were detailed in Tables 4, 5 and 6.

**Table 4:**
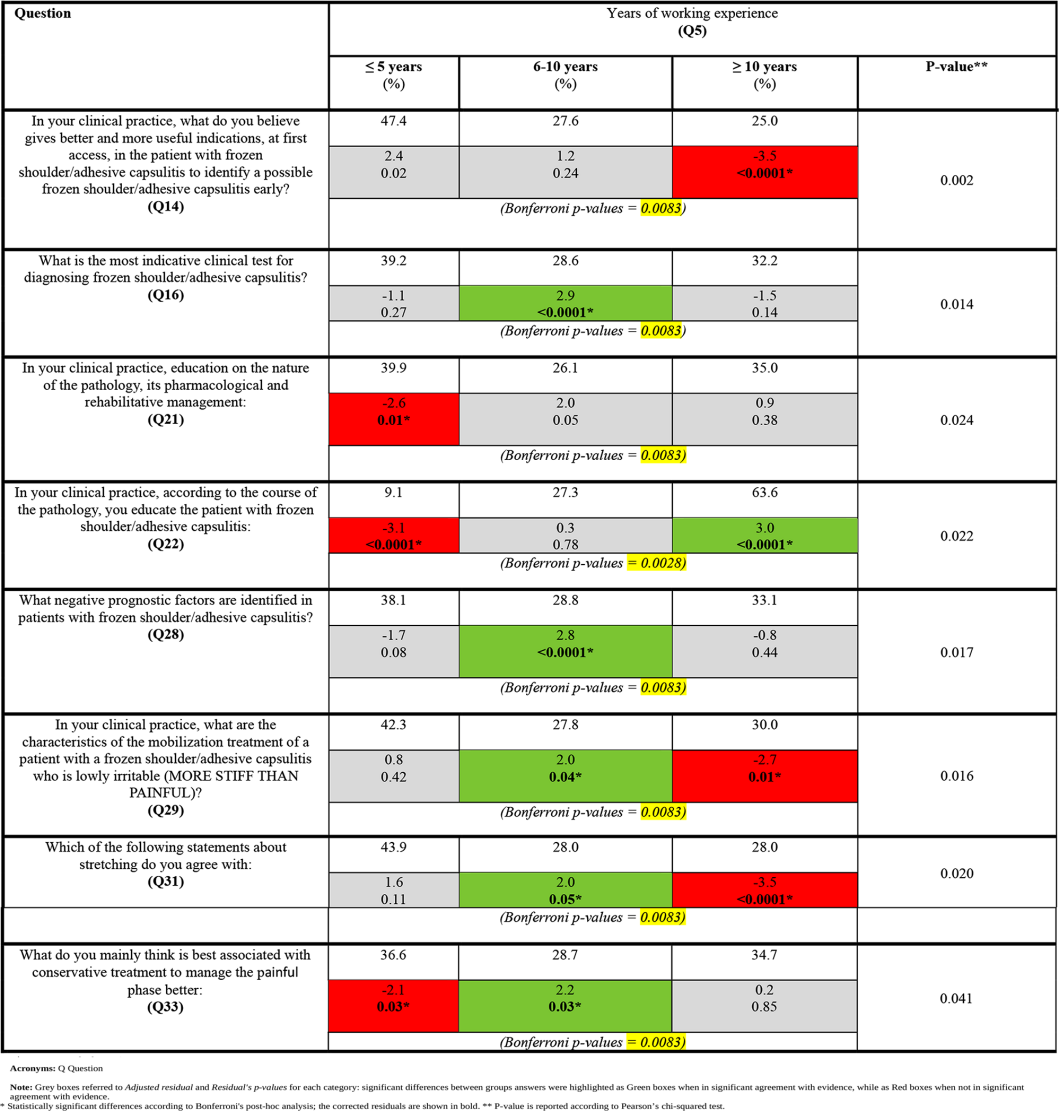
Inference between “years of work experience” and evidence-based recommended answers

**Table 5:**
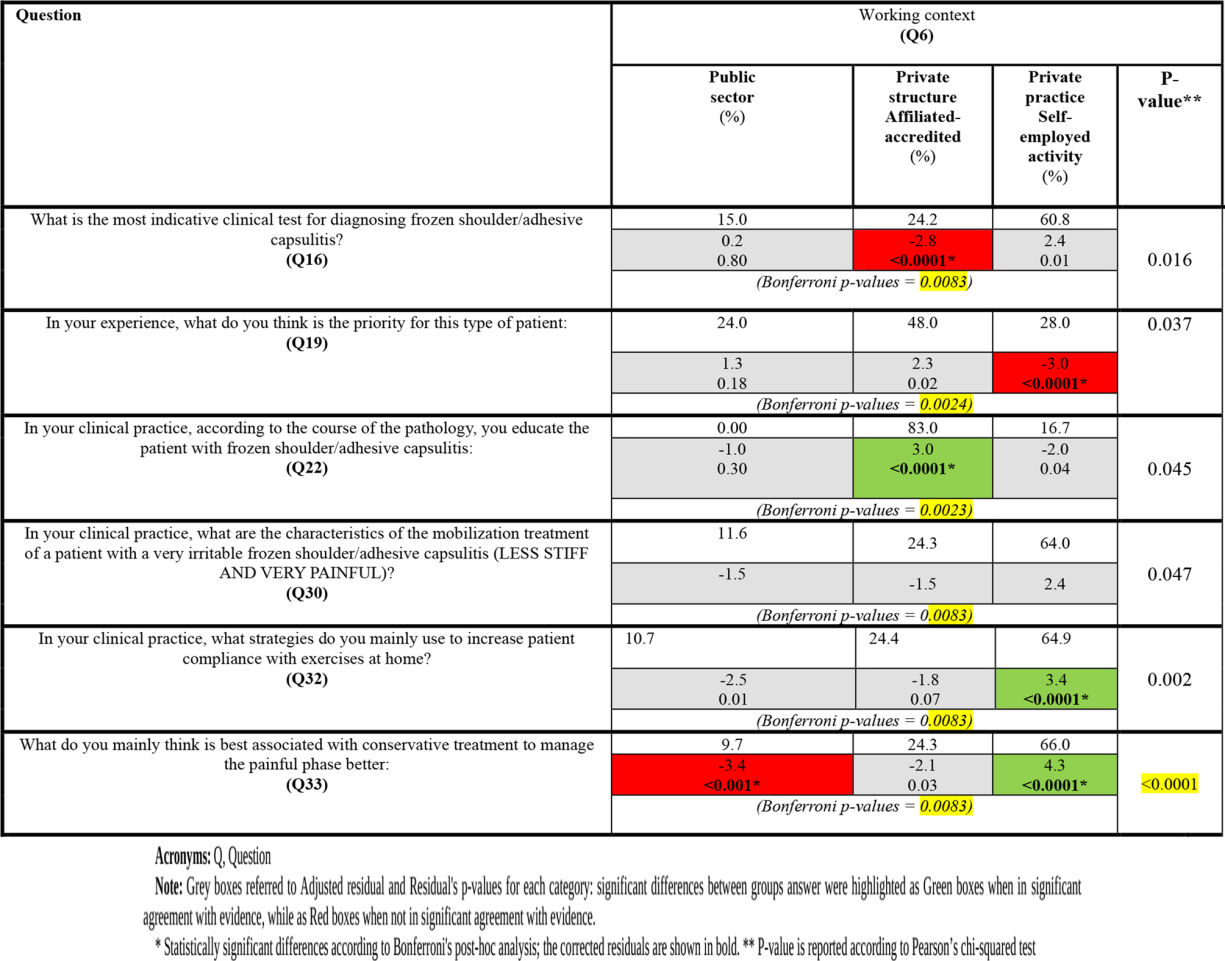
Inference between “working context” and evidence-based recommended answers

**Table 6:**
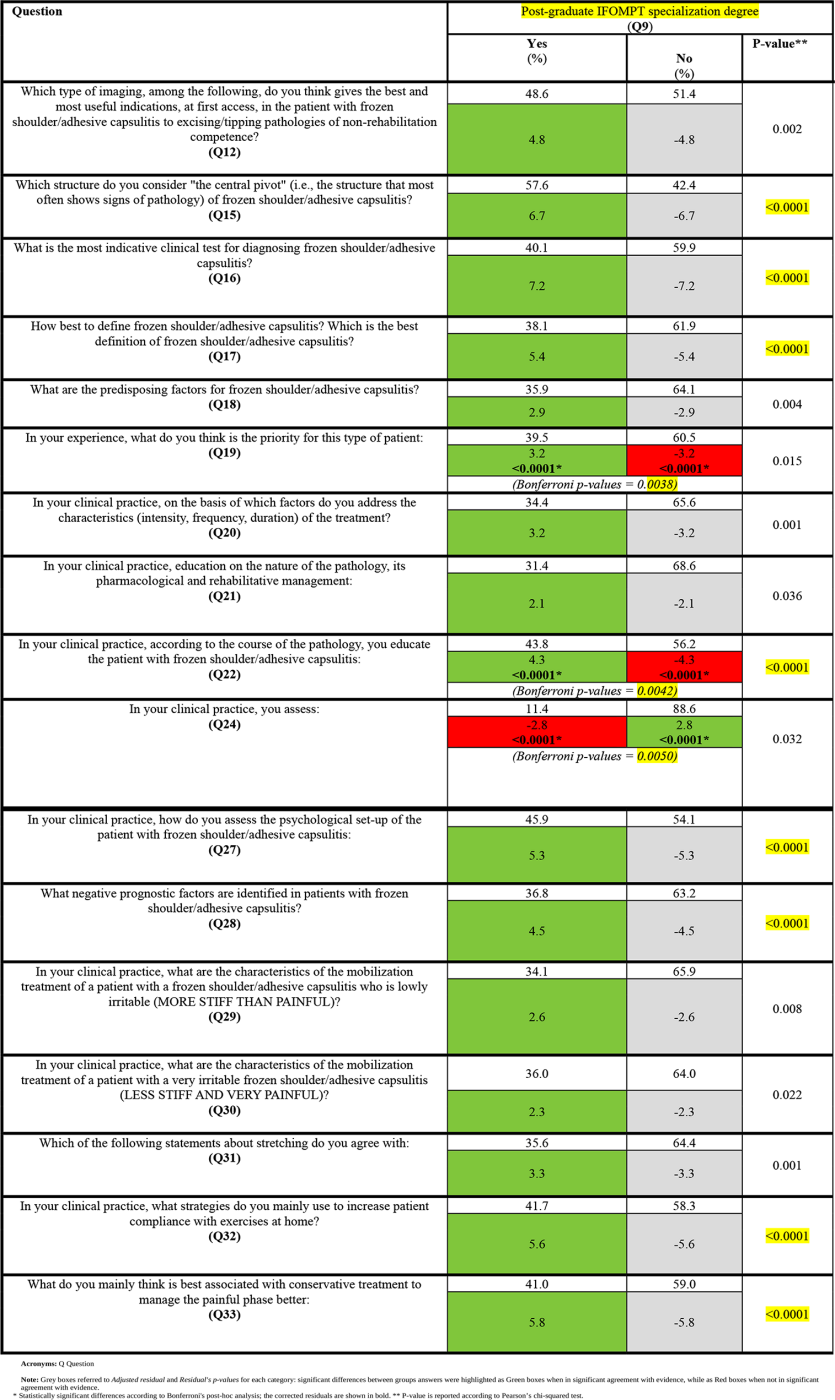
Inference between “Post-graduate IFOMPT specialization degree” and evidence-based recommended answers

## Discussion

The survey revealed that most respondents correctly defined FS [4]. However, there was diversity in explaining the evolution of FS, whether in three, two, or no phases, reflecting heterogeneity in the literature [38]. Current evidence suggests that the traditional chronological division of FS staging lacks interprofessional reliability and clinical relevance; in contrast, categorizing subjects based on high, moderate, and low irritability [3] has been recognized as better guidance for management, allowing for different treatment strategies. Unfortunately, respondents expressed a lack of confidence in these skills, particularly in managing very irritable/low stiff FS, showing a significant lack of consensus. These findings, consistent with previous surveys [24, 25], suggest a need for reflection among clinicians on the absence of evidence-based management guidelines. The newest introduction of the modern theoretical model categorizing FS based on irritability may justify the observed heterogeneity in responses.

To date, prognostic factors assessment has been judged more useful than diagnostic approach [39], and Italian PTs showed to acknowledge such clinical and anamnestic features, categorizing a-priori subjects that could have a worse prognosis.

In addressing the management of the painful phase of FS, most respondents considered it useful to incorporate pharmacological treatments or electrophysical agents. While pharmacological treatments are strongly supported for pain relief [15], electrophysical agents are not recommended, despite being widely used in the Italian clinical practice [22].

Stretching was reported as a valid strategy for improving the subject’s clinical situation as it improves the ratio of metalloproteinase to inhibitory tissues and tissue turnover [40–42], as most respondents stated. Conversely, some respondents indicated that stretching could increase the centimetric length of fibrotic structures, highlighting structure-centric and erroneous beliefs regarding the properties of manual therapy techniques [43, 44]. This is a matter of concern, as it might encourage clinicians to apply intense stretching, especially in the early stage when such practice is discouraged and could potentially exacerbate the inflammatory response by increasing the myofibroblast stimulation [5].

Lastly, as far as treatment is concerned, most Italian PTs used videos and mobile text messaging, demonstrating competence in enhancing therapeutic adherence and emphasizing the significance of long-term outcomes in FS subjects. Unfortunately, 22.8% of respondents do not use any particular strategy, possibly indicating a criticism of monitoring home exercise and engaging collaboration.

The identification of FS primarily relies on clinical evaluation, and radiographic imaging is recommended to exclude other pathologies, serving as a complementary diagnostic tool [5, 14, 45]. Our findings indicated that, during the initial assessment, Italian PTs considered X-ray, MRI, or a combination of both as the most useful diagnostic modalities. While this aligns with a previous survey from UK, United Arab Emirates and Korea [24, 25, 46], it’s worth noting that MRI should not be the initial choice for diagnosing FS, as it does not offer additional information beyond clinical examination and could incur unnecessary healthcare costs [47]. These imaging preferences may be influenced by the fact that Italian PTs are not authorized to prescribe diagnostic imaging scans due to legal constraints, and the academic curriculum may lack comprehensive training on imaging assessments.

The majority of Italian PTs identified the entire capsule as “the central pivot” of FS, and recognized bilateral comparison of ROM in external rotation with the arm by the side as the most indicative diagnostic clinical test, aligning with existing evidence [17].

However, the existing evidence suggests that “diagnostic maneuvers” should be supplemented by history taking, palpation, and X-ray examination [3, 4, 13] This is primarily because, in early FS, ROM limitation could be minimal and challenging to diagnose [24, 48] Consequently, FS is often diagnosed at a later stage when stiffness is well established [5, 45]. On this note, evidence indicates that ultrasound with Doppler may serve as a modality to differentiate early stage FS from rotator cuff tendinopathy. The enhanced vascularity and hypoechoic changes around the rotator cuff interval are significant specific indicators of early FS [49]. In the context of Italian physiotherapy practice, ultrasound imaging is allowed not for medical diagnosis purposes, but for a more comprehensive structural evaluation, improving clinical reasoning [50]. Once again, the divergence of PTs’ responses from evidence-based recommendations may be attributed to the fact that the interpretation of clinical examination and testing requires profound expertise and pathology-specific knowledge, which most respondents may not have obtained from their bachelor’s degree programme [51].

Less than half of the respondents accurately identified suggested clinical and anamnestic predisposing factors. One potential explanation is that most PTs completed a three-year BSc training, which may not offer adequate depth in comprehending the pathophysiology and epidemiology of specific clinical conditions. Moreover, there was a greater emphasis on diagnostic evaluation compared to prognostic evaluation [52, 53], emphasizing the importance of encouraging clinicians to identify prognostic factors from a biopsychosocial perspective. This approach has the potential to benefit individuals with FS through a timely, comprehensive, and collaborative strategy involving professionals beyond the scope of physical therapy [39].

Respondents underlined the importance of reassuring patients and addressing nightly pain as the top priorities for individuals with FS, aligning with previous research findings [17, 21, 54]. Moreover, advice and education about the pathology, coupled with simple strategies to modify occupational and recreational activities, were regarded as the primary antalgic strategies. This approach aimed to enhance adherence, reduce anxiety and depression, correct false pain beliefs, and alleviate feelings of uncertainty [5, 17, 21, 55]. Highlighting the significance of the psychological domain in FS subjects [39, 56], the survey results indicated that Italian PTs considered both anatomical factors (directly linked to the shoulder problem) and psychological aspects (such as fear, worry, anxiety, anger, distrust) to demonstrate competence in understanding the anatomical condition and being empathic and attentive to the psychological setup.

PTs preferred to work in a multidisciplinary team when competences of other professionals are required, consistently with what emerged from a previous survey [24] and more recent evidence suggestions [39, 57]. Italian PTs understood the value of multidisciplinary care in improving outcomes and the importance of a targeted/tailored approach that considers the bio-psychological aspects of managing FS. Unfortunately, most respondents were not accustomed to properly assessing psychological variables with dedicated patient-reported outcome measures; instead, extemporaneous non-standardized questions were preferred. This practice could be a limitation in evaluation, potentially leading to a lack of comprehensive assessment of the psychological domain and missing essential features for modifying care appropriately.

### Associations

This cross-sectional study also analysed the association between current evidence-based shared recommendations and different subgroups (Table 3).

PTs with less than six years and those with more than ten years of experience provided answers that did not align with the actual recommendations. A possible explanation for these results could be that the first mentioned had restricted knowledge, having only undertaken a three-year academic path and less expertise, and others could be less inclined and motivated to update their expertise.

Furthermore, PTs who worked in private practice or were self-employed significantly adhered to recommendations regarding increasing subject compliance and conservative management of the painful phase: probably, they may be more oriented toward following evidence to achieve results and customer loyalty [58, 59]; as well as for those with post-bachelor degrees, which tend to be significantly more competent in anatomical conditions and more adherent to recommendations about clinical tests and prognosis.

Similarly, PTs with higher university qualifications tended to align more with the literature, a trend consistent with previous investigations in Italy. PTs with post-graduate IFOMPT specialization degree showing significant agreement with evidence-based recommendations for most knowledge and management strategies. One possible explanation for this, is that such specialized courses are well-founded on evidence-based practice and adhere to international standards. [60]. This aligns with findings from other Italian surveys as well [26, 61]. Moreover, working in a direct access setting, managing multiple FS cases, or having 6 to 10 years of experience could imply greater knowledge, competence, and adherence to evidence-based clinical practice.

However, further prospective studies are needed to better evaluate these assumptions.

### Study limitations

The main limitation of this study was the categorization of the patients managed by PTs, as the first category was set at “≤ 2”, which implicitly could include the possibility of managing no patients at all.

Moreover, this survey investigated the PTs’ clinical practice through a non-a-priori validated questionnaire, which could weaken the robustness of the results. However, this survey represents the *first attempt* to understand the preferences, beliefs, and clinical practice of PTs regarding FS, and it could serve as a baseline for future investigations.

Additionally, our survey mostly recruited young and less experienced PTs, and the total sample was less than 1% of the total Italian PTs (*n* = 69,848). These characteristics could further weaken our results. However, the present sample remains one of the largest samples recruited in musculoskeletal field surveys in Italy.

Lastly, the survey’s administration through electronic devices and social media might have excluded PTs who are not familiar with these means. However, in today’s context, these communication channels are widely used among healthcare professionals. It’s worth noting that the participation of PTs with post-graduate IFOMPT specialization degrees was limited, representing a minority within the entire national scenario.

## Conclusion

This cross-sectional study highlights the preferences in clinical practices among Italian PTs for FS rehabilitation in comparison to evidence-based recommendations. Noteworthy, diagnostic imaging, clinical assessment, identification of predisposing factors, staging education, and mobilization modalities were areas where the PTs practice mostly diverged from the evidence’s suggestions. PTs with post-graduate IFOMPT specialization degrees, those with 6 to 10 years of clinical experience, and those working in private practice demonstrated greater appropriateness in terms of knowledge, competence, and adherence to evidence-based clinical practice.

### Electronic supplementary material

Below is the link to the electronic supplementary material.

Supplementary Material 1

Supplementary Material 2

## Data Availability

No datasets were generated or analysed during the current study.
