# Supplementary material for: Rehabilitation in subjects with frozen shoulder: a survey of current (2023) clinical practice of Italian physiotherapists
Source: BMC Musculoskelet Disord. 2024 Jul 23;25:573. doi: 10.1186/s12891-024-07682-w (PMC11265321; doi:10.1186/s12891-024-07682-w)
Supplement: Supplementary file 1 — Supplementary Material 1 [file 12891_2024_7682_MOESM1_ESM.docx]

**APPENDIX 2 – THE SURVEY**

**REHABILITATION IN SUBJECTS WITH FROZEN SHOULDER: A SURVEY OF CURRENT (2023) CLINICAL PRACTICE OF ITALIAN PHYSIOTHERAPISTS**

* it indicates a mandatory question

**SECTION A - DEMOGRAPHIC DATA**

1. Sex *

Mark only one option

- Man
- Female

1. Working area: *

Mark only one option

- Northern Italy
- Central Italy
- Southern Italy

1. Age: *

Mark only one option

- ≤ 25 years
- 26-35 years
- 36-45 years
- 46-55 years
- ≥ 56 years

1. Years of work experience: *

Mark only one option

- ≤ 5 years
- 6-10 years
- 11-15 years
- 16-20 years
- ≥ 21 years

1. Which work context are you most involved in: *

Mark only one option

- Public Hospital
- Private structure/affiliated-accredited
- Private practice/self-employed activity
- Home-based activity

1. Area of work which you are most engaged in: *

Mark only one option

- Musculoskeletal
- Sports
- Geriatric
- Neurology
- Other (Cardiological, Respiratory, Paediatric)

1. University education (select the highest degree acquired): *

Mark only one option

- Bachelor’s Degree in Physiotherapy
- Master’s Degree/Master of Science
- PhD

*PhD*, Doctor of Philosophy

1. Did you obtain a Level I Master’s Degree in Manual Therapy IFOMPT? *

Mark only one option

- Yes
- No

*IFOMPT*, International Federation of Orthopaedic Manipulative Physical Therapists

1. Average of working hours per week: *

Mark only one option

- 0-10
- 11-25
- 26-35
- 36-45
- ≥ 46

1. Number of patients with Frozen Shoulder treated, on average, per month: *

Mark only one option

- ≤ 2
- 3
- 4
- ≥ 5

**SECTION B - CLINICAL KNOWLEDGE AND EXPERTISE**

1. Which type of imaging, among the following, do you think gives the best and most useful indications, at first access, in the patient with frozen shoulder/adhesive capsulitis to excising/tipping pathologies of NON-rehabilitation competence? *

Mark only one option

- None, just the clinical examination
- X-ray
- MRI
- Ultrasound with Doppler
- X-ray and MRI
- MRI and Ultrasound with Doppler
- Ultrasound with Doppler and X-ray

*MRI*, Magnetic Resonance Imaging

1. Which type of imaging do you think gives the best and most useful indications, at first access, in the patient with frozen shoulder/adhesive capsulitis to early identify a possible frozen shoulder/adhesive capsulitis? *

Mark only one option

- None, just the clinical examination
- X-ray
- MRI
- Ultrasound with Doppler
- X-ray and MRI
- MRI and Ultrasound with Doppler
- Ultrasound with Doppler and X-ray

*MRI*, Magnetic Resonance Imaging

1. In your clinical practice, what do you believe gives better and more useful indications, at first access, in the patient with frozen shoulder/adhesive capsulitis to early identify a possible frozen shoulder/adhesive capsulitis? *

Mark only one option

- Anamnesis, X-ray, MRI
- Anamnesis, MRI, clinical tests such as “coracoid pain test”
- Anamnesis, physical examination of active and passive mobility, clinical tests such as “coracoid pain test”
- Anamnesis, physical examination of active and passive mobility, clinical signs such as “capsular pattern”

*MRI*, Magnetic Resonance Imaging

1. Which structure do you consider to be “the central pivot” (i.e. the structure that most often shows signs of pathology) of frozen shoulder/adhesive capsulitis? *

Mark only one option

- Long Head of the Biceps
- Coracohumeral ligament
- Tendon of the Supraspinatus
- The entire capsule
- Tendon of the Subscapularis
- None in particular

1. What is the most indicative clinical test for the diagnosis of frozen shoulder/adhesive capsulitis :*

Mark only one option

- Bilateral comparison of range of motion in flexion
- Bilateral comparison of range of motion in abduction
- Bilateral comparison of range of motion in internal rotation
- Bilateral comparison of range of motion in external rotation with the arm adducted

1. How best to define frozen shoulder/adhesive capsulitis? Which is the best definition of the frozen shoulder/adhesive capsulitis? *

Mark only one option

- Shoulder pathology characterized by night and day pain, with reduction in active and passive range of motion, especially in abduction in the coronal plane, the changes in which must remain stable for at least one month
- Shoulder pathology characterized by night and day pain at rest, with reduction of active and passive range of motion especially in external rotation with abducted arm. Furthermore, changes must remain stable for at least one month or worsen
- Shoulder pathology characterized by night and day pain at rest and in motion, with reduction of active and passive range of motion especially in external rotation with the arm adducted. Furthermore, changes must remain stable for at least one month or worsen
- Shoulder pathology characterized by night and day pain, with reduction of active and passive range of motion especially in sagittal plane flexion. Furthermore, the changes must remain stable for at least one month

1. What are the predisposing factors for the occurrence of frozen shoulder/adhesive capsulitis? *

Mark only one option

- Dysmetabolic diseases, age between 40 and 65, sedentary lifestyle, previous frozen shoulder, overweight, neurological and cardiopulmonary comorbidities
- Age between 40 and 65, previous frozen shoulder, neurological and cardiopulmonary comorbidities
- Dysmetabolic diseases, age between 40 and 65, hyperactivity, female sex, comorbidity
- Dysmetabolic diseases, age between 50 and 60, male, musculoskeletal morbidity

1. In your experience, what do you think is the priority for this type of patient: *

Mark only one option

- Daytime pain management
- Recovery of full range of motion
- Nocturnal pain management
- Restorative sleep recovery
- Recovery of autonomy (driving, dressing…)
- Functional recovery related to work activities, hobbies and social role
- Being reassured about one’s condition

1. In your clinical practice, on the basis of which factors do you address the characteristics (intensity, frequency, duration) of the treatment? *

Mark only one option

- Level of daytime pain, level of stiffness, number of sessions
- Relationship between active/passive ROM, level of night and day pain
- Level of day and night pain, active/passive ROM ratio, presence of pain related to range of motion
- Presence of pain related to range of motion, active/passive ROM ratio, presence of daytime pain
- Standard protocol for capsulitis

*ROM*, Range of Motion

1. In your clinical practice, education on the nature of the pathology, its pharmacological and rehabilitative management: *

Mark only one option

- It is an aspect that I often overlook as not interesting/useful for the patient
- It is a transversal intervention throughout the rehabilitation process, focused on the psychological component management
- It is a transversal intervention throughout the rehabilitation path, focused both on the psychological component and the pain portion management
- It is an aspect I consider not very important for rehabilitation management

1. In your clinical practice, according to the course of the pathology, you educate the patient with frozen shoulder/adhesive capsulitis: *

Mark only one option

- Evolution in 3 phases (in freezing-frozen-thawing), in detail
- Evolution in 2 phases (pain dominant or stiff dominant), in detail
- Evolution in 4 phases
- Evolution without specifying any phase
- With superficial explanations on this issue
- I do not consider it useful to provide this kind of explanation to the patient

CLINICAL KNOWLEDGE AND EXPERTISE

1. In your clinical practice, you usually manage the patient with frozen shoulder/adhesive capsulitis:

Mark only one option

- Independently
- In cooperation with the physician (orthopaedic, physiatrist)
- In cooperation with the psychologist
- In cooperation with the algologist
- In multidisciplinary teams, when the expertise of other professionals is required

1. In your clinical practice, you assess: *

Mark only one option

- Mainly anatomical aspects purely related to the shoulder problem (range of motion, pain, extent of stiffness)
- Ubiquitously the anatomical aspect purely linked to the shoulder problem and the psychological set-up (fear, worry, anxiety, anger, distrust...) linked to the shoulder problem
- The psychological rather than the anatomical aspect
- The anatomical rather than the psychological aspect

1. In your clinical practice, when dealing with a patient with frozen shoulder/adhesive capsulitis you tend to be predominantly: *

Mark only one option

- Empathetic and interested in building a relationship of trust
- Competent about the pathoanatomical condition, more than anything else
- Competent about the pathological condition, but equally empathic/attentive to the psychological set-up
- Exclusively attentive to the pathological condition

1. Based on your knowledge about the prognosis of the patient with frozen shoulder/adhesive capsulitis, which of the following statements do you consider to be the most correct? *

Mark only one option

- The patient always recovers 100%, net of rehabilitation efforts and prognostic factors
- Rehabilitation is often ineffective and not sufficient for optimal recovery and full patient’s satisfaction
- The natural pathology course ends with a “restitutio ad integrum”, without leaving any trace
- Coded factors that suggest to me, a priori, that the patient will be more unlikely to recover

1. In your clinical practice, how do you assess the psychological set-up of the patient with frozen shoulder/adhesive capsulitis: *

Mark only one option

- With validated measurement scales investigating catastrophizing, fear avoidance, anxiety and depression
- With extemporaneous, subjectivized and non-standardized questions
- With a history interview
- I do not assess the psychological setting of the patient

1. What are the negative prognostic factors identified in patients with frozen shoulder/adhesive capsulitis? *

Mark only one option

- Age > 60 years, thyroid disease, unilaterality of clinical presentation, less intense symptoms at onset
- Diabetes, hypothyroidism, external rotation at adducted arm > 0, more intense symptoms at onset
- Age < 60 years, external rotation at 0° adducted arm, diabetes and thyroid disease, bilaterality of clinical presentation, worse symptoms at onset
- Age, diabetes, hyperthyroidism, unilaterality of clinical presentation

1. In your clinical practice, what are the characteristics of the mobilization treatment of a patient with a frozen shoulder/adhesive capsulitis lowly irritable (MORE STIFF THAN PAINFUL)? *

Mark only one option

- Mobilization below the pain threshold, in any direction, not very intense, at the end of the range of motion
- Intense degree mobilization, posterior direction, moderately painful (approx. 6/10 NPRS) at the end of the range of motion
- Painful mobilizations, in any direction, not very intense, not at the end of the range of motion
- Non-painful mobilizations, intense degree, not at the end of range of motion, in posterior direction

*NPRS*, Pain Numeric Rating Scale

1. In your clinical practice, what are the characteristics of the mobilization treatment of a patient with a very irritable frozen shoulder/adhesive capsulitis (MORE PAINFUL THAN STIFF)? *

Mark only one option

- Mobilization below the pain threshold, in any direction, not very intense, at the end of the range of motion
- Intense degree mobilization, posterior direction, moderately painful (approx. 6/10 NPRS) at the end of range of motion
- Painless mobilization, in any direction, not very intense, not at the end of range of motion
- Non-painful mobilizations, intense degree, not at the end of range of motion, in posterior direction

1. Which of the following statements about stretching do you agree with: *

Mark only one option

- Increases the centimetric length of fibrotic structures
- Improves the balance between metalloproteinase and tissue inhibitors of metalloproteinase
- Worsens tissue turn over
- Always increases pro-inflammatory expression

1. In your clinical practice, what kind of strategies do you mainly use to increase patient compliance with exercises at home? *

Mark only one option

- Mobile phone videos and messages for motivational/educational purposes
- Illustrative booklet
- Diary
- None in particular
- I do not administer exercises at home

1. What do you mainly think is best associated with conservative treatment to better manage the painful phase: *

Mark only one option

- Physical therapy (laser therapy, diathermy, ultrasound, shockwaves)
- Cortisone therapy (oral or infiltrative)
- De-tensioning massage therapy
- Non-steroidal anti-inflammatories
