# Supplementary material for: Rehabilitation in subjects with frozen shoulder: a survey of current (2023) clinical practice of Italian physiotherapists
Source: BMC Musculoskelet Disord. 2024 Jul 23;25:573. doi: 10.1186/s12891-024-07682-w (PMC11265321; doi:10.1186/s12891-024-07682-w)
Supplement: Supplementary file 2 — Supplementary Material 2 [file 12891_2024_7682_MOESM2_ESM.docx]

**APPENDIX 1 – INVITATION LETTER**

**Knowledge and Expertise of Italian physiotherapists regarding the 'frozen shoulder/Adhesive Capsulitis’ pathology**

Presentation of the project and participant's consent to complete the questionnaire

Welcome to the project designed to investigate the knowledge and treatment approaches of Italian physiotherapists concerning patients with Frozen Shoulder/Adhesive Capsulitis.

*What is the objective of this project?*

Some physiotherapists of the Master in Science and Practice in Musculoskeletal and Rheumatology Physiotherapy at the University of Molise have developed a questionnaire to investigate the knowledge and treatment methods of Italian physiotherapists in dealing with patients with Frozen Shoulder/Adhesive capsulitis.

*What does participation consist in?*

Your participation is entirely voluntary and anonymous. It involves completing a brief questionnaire, requiring approximately 10-15 minutes. There are no right or wrong answers; this is an opportunity to share your impressions, beliefs, and clinical practices.

*Who can participate?*

Participation in this study is open to individuals holding a degree in physiotherapy and currently practicing in Italy.

*Should you have any questions*, please feel free to contact a team member via email: [assenzaraffaele@gmail.com](mailto:assenzaraffaele@gmail.com)

Before providing your consent to participate in the questionnaire, it is important to note that your involvement will be strictly confidential, and no information about you will be used in any way that reveals your identity. The questionnaire is anonymous and voluntary, and there are no incentives planned for completing the survey.

* it indicates a mandatory question

Do you want to participate in this study? *

The participant who willingly chooses to take part in the study must expressly grant consent by clicking the "YES" button, confirming acceptance.

Mark only one option

- Yes, I want to be directed to the questionnaire
- No, Thanks
